# Ketamine for Bipolar Depression: Biochemical, Psychotherapeutic, and Psychedelic Approaches

**DOI:** 10.3389/fpsyt.2022.867484

**Published:** 2022-05-23

**Authors:** Raquel Bennett, Christian Yavorsky, Gary Bravo

**Affiliations:** ^1^KRIYA Institute, Berkeley, CA, United States; ^2^Valis Bioscience, Berkeley, CA, United States

**Keywords:** bipolar, depression, intramuscular, ketamine, psychedelic, psychotherapy, racemic, suicidal

## Abstract

Bipolar disorder (type 1) is a serious and chronic psychiatric illness that can be difficult to treat. Many bipolar patients have refractory depressive episodes. Racemic ketamine, a glutamate modulator with prominent dissociate and psychedelic properties, has been demonstrated to have rapid acting antidepressant and anti-obsessional effects which may be useful for treating the symptoms of bipolar depression. Most of the existing research literature on unipolar and bipolar depression has looked at racemic ketamine in the sub-psychedelic dose range given by infusion as a stand-alone treatment (without concurrent psychotherapy). This article expands on the existing research by articulating three different paradigms for ketamine treatment: biochemical, psychotherapeutic, and psychedelic. The authors use composite clinical vignettes to illustrate different ways of working with ketamine to treat bipolar depression, and discuss a variety of clinical considerations for using ketamine with this population, including route, dose, frequency, chemical mitigators, and adverse events. Note that the conceptual paradigms could be applied to any ketamine treatment, with broad applicability beyond bipolar treatment.

## Introduction

Bipolar disorder (type 1) is a serious and chronic mood disorder that affects nearly 3% of the population globally (1). It is associated with high rates of disability, suicidal ideation, and completed suicides (2). Conventional treatment for this population typically includes oral antidepressant and mood stabilizing or antipsychotic medication, and possibly anxiolytic or hypnotic medications (3). Even with these medications, many patients with bipolar disorder (type 1) struggle with refractory and recurrent depressive episodes (4). Further, the medications can cause bothersome side effects which interfere with patient compliance. In other words, the medications that are widely used at this time may not sufficiently meet the needs of these individuals (5).

Ketamine treatment may be helpful in addressing the unmet needs of patients who are living with bipolar disorder (type 1). Scientific studies have demonstrated that racemic ketamine is clinically effective in reducing the symptoms of bipolar depression (6, 7). It works rapidly, often taking effect in a matter of minutes. It is generally well tolerated and has a short half-life, which translates into fewer side effects than traditional antidepressant medications. Most of the existing research to date on the use of racemic ketamine for depression (unipolar and bipolar) has looked at a sub-psychedelic dose of ketamine given by infusion as a stand-alone treatment, without concurrent psychotherapy [See (8) for a meta-analysis of previous research studies].

However, ketamine is a versatile tool which can be used in variety of ways (9). Here we articulate three paradigms for ketamine treatment: biochemical, psychotherapeutic, and psychedelic. The biochemical model focuses on the biological effects of the medication alone. The psychotherapeutic model utilizes ketamine as a lubricant for the psychotherapy process. The psychedelic model makes use of the prominent dissociative and psychedelic properties of ketamine to purposefully induce a temporary altered state of consciousness for psychospiritual exploration.

Using composite clinical vignettes, we illustrate the three approaches to ketamine treatment for refractory depression in patients with bipolar disorder (type 1). These composite cases are based on patients in our clinical practices (10, 11). We also describe a variety of clinical considerations for using ketamine with this population, including notes on route, dose, frequency, chemical mitigators, adverse events, etc. These observations were informed by our clinical practice, consultation with our colleagues, conference material, and patient reports, and should be viewed as preliminary observations from the field which necessitate further research.

## Clinical Vignettes

### Biochemical Paradigm

*Alex was an artistic 21-year-old male college student with a slender build (6'4“ and 180 lbs). He was diagnosed in childhood with Marfan Syndrome and aortic dilation which did not require surgery. Alex, who was studying architecture and art history, began struggling with periods of elation, risk-taking and withdrawn depression. He had several encounters with the local police due to disorderly conduct. Eventually he was diagnosed with bipolar disorder (type 1) by the psychiatrist at the university health center, and started on lithium and escitalopram*.

*During an intense period of immobilizing depression, Alex's parents brought him to our mental health clinic for consultation. He presented with severe depression including apathy, poverty of speech, and ruminative suicidal ideation without a specific plan. We assessed him and did some psychological preparation him, then we referred Alex to an anesthesiologist for ketamine treatment because of concerns about his cardiac health. Alex received six ketamine infusions (0.5 mg/kg per infusion) in a 3-week period, in addition to weekly psychotherapy. He reported that each ketamine infusion had some similarities to light alcohol intoxication. Alex began showing signs of improvement following the fifth infusion: he became more verbal and engaged in therapy and expressed a desire to complete his education. Alex continued in individual psychotherapy while he completed his senior year, where he worked on recognizing prodromal symptoms, eating and sleeping regularly, time management, and setting limits*.

### Psychotherapeutic Paradigm

*Bijan was an ambitious 47-year-old male businessman (5'9” and 210 lbs). He had three children and was divorced. Bijan had been diagnosed with bipolar disorder (type 1) in his twenties during a psychiatric hospitalization for psychotic and suicidal behavior. He had taken a variety of psychiatric medications over the years, but suffered with significant side effects (including digestive issues, weight gain, and erectile dysfunction) which caused him to stop taking his medications periodically, which in turn would cause his symptoms to intensify*.

*Bijan was seen for weekly individual psychotherapy and bi-weekly medication management. He was stabilized on bupropion and quetiapine, which did not provide complete symptom relief, but which had a tolerable side effect profile. After several months of treatment, we added three sessions of ketamine-facilitated psychotherapy using injectable ketamine (0.7 mg/kg per injection) spaced several weeks apart. Bijan said that the medicines felt pleasant and relaxing. His ketamine sessions felt like an amplification of his regular talk therapy material: he began to wonder about the impact of his illness on his career and his marriage, he realized that he behaved aggressively or erratically when he was off his medications, and he realized that he had a lot of unexpressed feelings about his divorce. He reported that the ketamine sessions helped to stave off acute depression and gave him more space to think and feel*.

### Psychedelic Paradigm

*Charlie was an articulate and affable 64-year-old male professor (5'10 and 170 lbs). He was the author of several textbooks, a popular academic mentor, and enjoyed doing outdoor sports and playing music in his free time. He reported that he had a long history of depressive episodes that began in childhood. As a young man, Charlie had bouts of over-spending, gambling, and hypersexual behavior, and was diagnosed with bipolar disorder (type 1) in his early thirties. At that time, he started taking valproic acid (with PRN olanzapine) and going to psychotherapy. A few years later, he began practicing mindfulness meditation to help him cope with his mood swings and cravings*.

*Charlie came to us for psychotherapy to explore his feelings about retirement, concerns about aging, and strategies for managing his recurrent depressive episodes. He stated that he was plagued with feelings of inadequacy for as long as he could remember and believed that he was fundamentally damaged in some way. Charlie was seen for approximately 6 months of psychotherapy with 1-2 meetings per week. His mood began to deteriorate following the unexpected death of a close colleague. Charlie was given two sessions of injectable ketamine in the psychedelic dose range which were fully dissociative and resulted in a loss of interest in external stimuli. except for hypersensitivity to sound. In the first session (1.1 mg/kg), he saw himself on a floating conveyor belt, passing pedestals which represented important accomplishments and challenges in his life. The conveyor belt ended at a wall of light; he sensed that there was something important beyond the wall. Four weeks later, Charlie had the second session (1.4 mg/kg). He reported that his body dissolved, and his awareness was released from his body. His spirit floated in a pool of rainbow light and had a profound sense of peacefulness and wellbeing to the core. Charlie was able to remember and make use of this feeling in the months of psychotherapy that followed*.

## Discussion

### Differential Diagnosis

Before commencing ketamine treatment with any patient, it is important to establish and confirm the patient's clinical diagnosis, as the differential diagnosis guides the treatment plan. We have encountered many patients over the years who were misdiagnosed. For example, we have frequently seen patients who were previously diagnosed with refractory unipolar depression, depression with anxiety, depression with attention deficit disorder, or depression with psychotic features, but who were better understood as having undiagnosed (and untreated) bipolar disorder. We have also seen patients who were previously diagnosed with bipolar disorder who were better understood as having adjustment disorder, sleep disturbance, substance use disorder, or complex PTSD. Sometimes these are overlapping or co-morbid conditions (12).

### Trauma

Of special interest here is the potential overlap between bipolar spectrum disorders and complex post-traumatic stress disorder (CPTSD), which can look very similar upon initial presentation (13). While ketamine undoubtedly can be helpful for some patients with PTSD or CPTSD (14), we are also aware of numerous cases where patients were unexpectedly thrown into revisiting traumatic material during their ketamine treatment and were distressed by the experience. At this point, we have reservations about giving a powerful dissociative chemical such as ketamine to patients with a history of dissociative trauma, especially in the absence of substantial psychological support. Further, for patients who have both a bipolar spectrum disorder and PTSD or CPTSD, we have found that it is useful and important to stabilize the organic mood disorder with medications as much as possible first, before delving into an exploration of traumatic material. Working with traumatic memories can be destabilizing, and can feel unbearable when amplified by endogenous mood instability.

### Mood Stabilizing Medication

It is advisable for bipolar patients to have a mood stabilizing or antipsychotic medication in their regimen before beginning ketamine treatment. Part of the purpose is to provide as much symptom relief and overall mood stability as possible. This is also important to prevent elevations into hypomania or mania (affective switching) following ketamine administration (15, 16). However, prescribing these kinds of medications is a delicate art to achieve the clinically significant effect without excessive sedation or cognitive impairment.

### Psychosis

Historically, there has been concern about administering ketamine to patients who have a history of psychosis. This concern can be traced back to research that was done at Yale in the 1990s, where ketamine was used to induce altered states of conscious for the purpose of studying schizophrenia (17). The next generation of ketamine researchers went on to erroneously and pejoratively apply the term “psychotomimetic” to any expansive or mystical-type experiences that the subjects reported in the studies that were conducted on ketamine infusion for depression (18). This error, which was replicated for many years, represents a fundamental misunderstanding of the nature of psychedelic experience. It is unfortunate that this conflation of “psychotic” and “psychedelic” has caused some clinicians and researchers to exclude patients with refractory bipolar depression from ketamine. We think this exclusion criteria is largely unwarranted, especially when patients are stabilized with medication (see above). Further, new research is investigating ketamine as a possible treatment for the negative symptoms of schizophrenia (19).

### Chemical Mitigators of Ketamine

There are several chemicals which are potent ketamine mitigators: benzodiazepines (20, 21), alcohol (22), opiates and opioids (23), and barbiturates. These substances clearly attenuate the therapeutic benefit of ketamine treatment for most patients, as well as the subjective experience. Benzodiazepines are of particular interest here, as they are often prescribed to bipolar patients. There are several different strategies that the psychiatrist might employ in this situation, such as considering the feasibility of reducing the patient's benzodiazepine use, increasing the ketamine dose slightly to compensate for the presence of the benzodiazepines, and/or discussing frankly with the patient about the possible interaction. We have observed in multiple cases that ketamine treatment is greatly enhanced by stopping or reducing benzodiazepine use for several days prior to ketamine administration, but we are also aware of the discomfort and dangers associated with reducing or stopping benzodiazepines. Therefore, it is a matter of clinical judgment about how to approach this dilemma.

There are a number of other chemicals that may be mild ketamine mitigators, including antipsychotic medications (24), lamotrigine (25), cannabis (26), and kava. (There is also a question based on clinical observation of a small number of patients about possible interactions with NSAID pain relievers and/or antihistamines (27). In our clinical experience, patients vary tremendously in their sensitivity to these interactions. The amount and timing of exposure to any of these chemicals also likely plays a role. Whenever a patient does not respond as robustly as desired to ketamine treatment, we look for possible exposure to a chemical mitigator. With respect to mitigating medications, we recommend similar strategies and considerations as for benzodiazepines (see above), keeping in mind the potential risks and benefits of altering the patient's medication regimen.

### Contraindications

A complete discussion of all potential chemical interactions and medical contraindications for ketamine treatment is beyond the scope of this article, but it is important to note concern about combining ketamine with substances that cause hypertension, sedation, or respiratory depression.

### Substance Abuse

A substantial portion of bipolar patients also have substance use issues (28). We are very wary of giving ketamine to patients who are using other drugs outside of their prescribed regimens, although we have colleagues who are working with ketamine as an adjunctive treatment for substance use disorders, where the substance use is the focus of clinical treatment (29). We are aware that some of our colleagues require urine screens before ketamine administration, for the patient's safety and/or the provider's liability.

### Different Approaches to Ketamine Treatment

Racemic ketamine is a powerful and versatile tool that can be utilized in a variety of ways in clinical treatment for certain mental health indications (30). Currently there is much disagreement in the field about “the right way” to use ketamine in psychiatry and psychotherapy (31). In our experience, we have found that different individuals appear to benefit from different approaches to ketamine treatment at different points in the arc of their illness management.

#### Biochemical Paradigm

The “biochemical” model focuses on the biological effects of ketamine and treats ketamine's prominent dissociative/psychedelic properties as a problematic side effect (32). Further, in this model, little attention is paid to the patient's mental state (“set”) or the environment (“setting”) (33, 34). The patient is viewed as a passive vessel which receives the pharmaceutical. Much of the early research on ketamine treatment for depression was done in this paradigm, typically with 0.5 mg/kg of ketamine administered by intravenous infusion over 40 min. In this paradigm, patients often receive six ketamine infusions within the span of 2 or 3 weeks (35). The ketamine infusions are spaced relatively close together to create a cumulative series. It is essential to understand that the need for six treatments is predicated on getting a low dose of ketamine, which is sub-psychedelic by design.

We think that this approach is well-suited to patients (such as Alex) who have cardiac issues or who are medically complicated. In our opinion, it is prudent to send those patients to an anesthesia provider in our community who can provide a very high level of medical monitoring and care, if needed. In addition, patients who are living with mood disorder and physical pain may be good candidates for treatment with an anesthesia provider because of their expertise in pain management. Sometimes, patients choose to see an anesthesia provider in their community for logistical reasons.

It is important to note that many anesthesia providers offer ketamine infusions for mood disorders as a stand-alone treatment, without recommending or requiring concurrent psychotherapy or behavioral strategies. We have concerns about this practice; we believe that patients who are being treated for a mental health reason should have consultation and/or treatment with a mental health professional (36).

In our vignette, Alex got both ketamine infusions and separate but concurrent psychotherapy (asynchronous treatment), and then continued in psychotherapy after the termination of the ketamine treatment. This pattern of treatment is typical of the patients in our clinical practices who are referred out for ketamine infusions. Research done by Wilkinson et al. (37) and supports this asynchronous combination of modalities, and clearly further research is needed.

#### Psychotherapeutic Paradigm

The “psychotherapeutic” model utilizes ketamine as a lubricant or a catalyst for the psychotherapy process, with an emphasis on the verbal expression and emotional metabolism of the patient's thoughts and feelings during the ketamine administration (synchronous treatment). Psychotherapy is an integral part of this approach. (This approach has some conceptual similarities to the studies on using MDMA to treat PTSD) (38). This type of work can be done with 0.3–0.9 mg/kg of bioavailable ketamine, given by any route that the provider prefers (e.g., intramuscular injection, compounded transbuccal lozenge, nasal spray, intravenous infusion) (39). This dosing strategy is also sub-psychedelic by design. While there may be some alteration of consciousness during this kind of treatment, it is important to keep the dose low enough so that the patient is able to articulate their ideas and engage in meaningful self-reflection.

This approach is well-suited to patients (such as Bijan) who are in an established psychotherapy relationship, and who are motivated to deepen their understanding of themselves and their difficulties. We have observed that patients who engage in ketamine assisted psychotherapy (KAP) tend to revisit material that they have explored previously in psychotherapy, and they often spontaneously experience a consolidation of their insight or commitment to behavioral change. With the help of on-going psychotherapy, these patients can be supported in implementing the changes that they envisioned. Some patients benefit from a few KAP sessions, while others need a longer series. We tend to space KAP sessions 3 to 6 weeks apart, with regular talk therapy in between, to allow time to digest the psychological material, but we are aware that some of our colleagues are using KAP on a bi-weekly or weekly schedule on a time-limited basis with select patients (40) [See (41, 42) for further discussion of KAP].

#### Psychedelic Paradigm

The “psychedelic” model makes use of ketamine's prominent dissociative and psychedelic properties, and uses the medicine to intentionally induce a profound and temporary altered state of consciousness which is characterized by vivid, dream-like visions (43). This type of experience requires specialized psychological support before, during, and following the ketamine administration. The physical setting also contributes to the overall experience; many clinicians use ceremonial elements to separate the psychedelic session from regular daily life (44, 45). (This approach has some conceptual similarities to the studies on using psilocybin to treat end-of-life anxiety) (46). This kind of ketamine treatment is frequently done using an intramuscular injection in the dose range of 1.0–1.5 mg/kg (47). Higher doses (up to 2.0 mg/kg) are occasionally used, although we have not observed a stronger or more durable benefit in our patients, and the experiences tend to become more fragmented and/or amnestic (48). While early ketamine researchers were fearful of ketamine's psychedelic properties, subsequent research has suggested that the experience of awe (49) or other kinds of psychedelic experience during ketamine administration may be correlated with more potent antidepressant effects (50, 51) [See (52, 53) for further discussion on the use of psychedelic ketamine journeys in clinical treatment].

Not every patient is well-suited for full-blown dissociative and psychedelic experience at the beginning of ketamine treatment, or ever. It is helpful if patients (such as Charlie) have cultivated the ability to observe their own mental processes before beginning psychedelic treatment (e.g., through meditation or psychotherapy). Patients who have expressed a genuine curiosity about existential issues (e.g., the nature of reality) tend to find value in this kind of exploration. It is important to have a solid therapeutic alliance in place, which takes time and effort, before attempting this kind of treatment. Finally, there is a tendency in the current zeitgeist to idealize psychedelic experience. However, both the patient and the provider need to recognize that this is not a shortcut, and more medicine is not necessarily better medicine.

#### Different Approaches for Different Treatment Objectives

In the vignettes, all the bipolar patients received some combination of ketamine and psychotherapy, in addition to conventional oral pharmacotherapy. The first patient (Alex) represents a medically complicated young adult, and the focus of his treatment was to reduce the severity of his depressive symptoms. He also had psychotherapy that extended beyond his ketamine treatment, primarily to learn new skills that would help him to function. The second patient (Bijan) not only needed treatment for his refractory bipolar depression, but also had an openness to reflecting on the impact of his illness on his intimate relationships and professional life. In this case, the ketamine was used in service of facilitating personal insight, which lead to make concrete behavioral changes. The third patient (Charlie) needed treatment for his depressive spiral. His psychedelic ketamine experience allowed him to grapple with his feelings of defectiveness despite outward material success, and he did a deep piece of psychological work on his sense of self and relationship with his lifelong illness. It would appear that ketamine and psychotherapy together produced results which could not be achieved through ketamine or regular psychotherapy alone.

It is worth noting that when clinicians in the field talk about the treatment of bipolar patients (type 1), they often focus on the diagnosis phase and acute symptom management. However, it is useful to remember that patients who live with a lifelong and potentially debilitating illness often benefit from different kinds of support at different points in their lives: identifying the disorder, containing clinical symptoms, understanding the impact of their condition on daily living, and grieving for the losses that are inevitably associated with chronic illness (54).

### Route and Dose

In the vignettes above, the ketamine doses ranged from 0.5 to 1.4 mg/kg, and produced a range of subjective experiences. One patient received ketamine by infusion from an anesthesiologist, and the other patients received ketamine by injection from a physician in the presence of a psychotherapist. Note that in our clinical practices, we prefer to use injectable (intramuscular) ketamine because it is inexpensive, easy to administer, and has high bioavailability (55). The total ketamine dose can be divided into multiple injections, if desired. The route of administration and dose in ketamine treatment are independent variables; any dose of ketamine can be given by any route.

### Frequency in the Induction Phase

The induction phase is the initial phase of ketamine treatment. There is an inverse relationship between dose and frequency. Patients typically need to be seen more often for lower dose ketamine treatment, and the sessions need to be close together in time so that the effects are cumulative, until the initial series is completed. When patients receive a moderate or higher therapeutic dose of ketamine, the sessions need to be spaced out so that patients have time to process the material that arose.

### Time of Day

We have observed that ketamine treatment tends to interfere with sleep for our bipolar patients, although this phenomenon has not been formally researched to date. Specifically, many of our bipolar patients have reported difficulty falling asleep for up to twelve hours following ketamine administration, even with hypnotic medication in their regimen (56). This phenomenon seems particularly pronounced in bipolar patients who have the diurnal variation of depressed mood in the mornings and improved (or elevated) mood in the evenings (57), and ketamine dose may also be a factor (a higher dose of ketamine may be more disruptive to sleep than a lower dose). For this reason, we prefer to do ketamine sessions with our bipolar patients in the early part of the day, preferably before 12 noon, whenever possible.

### Synchrony

In all of the vignettes, the patients received ketamine paired with concurrent psychotherapy. In this context, “concurrent” means overlapping in a span of time, although not necessarily in the same session or location. Alex received asynchronous ketamine and psychotherapy, meaning that the infusion and psychotherapy appointments were separate. Bijan and Charlie received synchronous ketamine and psychotherapy, meaning that the ketamine was administered during the psychotherapy session. This is a useful distinction as the ketamine field expands.

### Frequency in the Maintenance Phase

One area of ketamine treatment that has not been well documented is maintenance treatment. Currently, many of our colleagues utilize a reactive model and wait for patients to relapse before offering another round of treatment. However, we know that bipolar disorder (type 1) is typically a lifelong, incurable, and cyclical illness. We wonder if a prophylactic model would make sense for this population, which would look like having ketamine treatment at regular intervals in an effort to maintain wellness and/or resolve refractory symptoms before they become clinically significant. Further research is needed to determine the appropriate intervals for this strategy, keeping in mind that excessive ketamine exposure may lead to cystitis (see below).

### Enantiomers of Ketamine

We prefer to use racemic ketamine in our clinical practices for a variety of reasons (58). Racemic ketamine is a generic medication in the United States. As such, it is broadly available as a medical supply, and it is extremely inexpensive. We can choose the route, dose, and frequency of treatment to suit the individual needs of our patients. Further, the two enantiomers of ketamine appear to have slightly different effects on people: the S-enantiomer (esketamine) appears to be more activating, and the R-enantiomer (arketamine) appears to be more sedating (59). We have had several patients who received esketamine and racemic ketamine treatment at different times, and they reported that esketamine felt too stimulating or “speedy” for them (60). We are tentatively hypothesizing that racemic ketamine treatment provides a good balance of the activating and soothing properties of ketamine, and that this balance appears especially well suited to treating bipolar patients, and that this may be different from patients with unipolar depression. From our perspective, the only drawback to choosing racemic ketamine for psychiatric treatment is that it is an off-label use at the current time.

### Side Effects and Adverse Events

As noted in other places, ketamine is generally well tolerated, but there are a number of potential side effects. Common transient or treatable side effects of ketamine treatment include elevated pulse and/or blood pressure, headache, nausea, anxiety, changes in vision, changes in muscle tension, and/or unwanted feelings of dissociation (61). Ketamine use over time has been linked to cystitis and bladder dysfunction (62, 63). Serious adverse events associated with ketamine treatment in a legal setting include respiratory distress and seizure (64).

### Suicidality

Many individuals with bipolar spectrum disorders experience suicidal ideation as a symptom of their illness. There is some evidence that ketamine treatment can be helpful in rapidly alleviating suicidal ideation in unipolar and bipolar patients, including studies on racemic ketamine infusions (65) and esketamine nasal spray (66). In our clinical practices, we have had success in alleviating suicidal ideation using racemic ketamine in a variety of dosing strategies and routes of administration. It sometimes works very quickly (<10 min following the introduction of ketamine), and the biochemical effect is often temporary (lasting a few hours to a few days) unless the ketamine treatment is paired with psychotherapy. Suicidal patients who respond robustly to ketamine treatment typically have chronic, ruminative, and/or ego dystonic suicidal ideation which has an obsessive quality to it. This observation raises a question as to whether the ketamine is acting on the self-harmful thought content or the ruminative thought process?

We have also observed that ketamine treatment occasionally increases suicidal ideation in a small fraction of severely depressed individuals (unipolar or bipolar), and this observation has been corroborated by a recent study of ketamine treatment in “real world” clinical treatment (67, 68). We are also aware of several completed suicides that occurred during or following a course of ketamine treatment (69). It is unclear why some patients feel significantly worse in response to ketamine treatment, but ketamine providers need to be aware of this possibility and be extremely cautious about prescribing ketamine for use outside of their direct clinical supervision. Further, individuals with chronic and refractory mental health conditions are inherently a high risk group for adverse psychiatric events, and ketamine is powerful psychoactive medicine that works differently than “traditional” antidepressant or mood stabilizing medications. For these reasons, it is imperative that clinicians who want to work with ketamine for mental health indications seek out specialized training.

## Conclusions

Bipolar disorder (type 1) is a difficult disease to treat. Here we added to the existing literature on this topic by offering our observations from the field on the use of racemic ketamine to treat patients with refractory bipolar depression. We discussed a number of practical considerations for working with this population, including the importance of the differential diagnosis at the beginning of treatment; information about the route of administration, dose, frequency, and timing of ketamine treatment; interactions with other medications; reasons to use racemic ketamine instead of a single enantiomer, and adverse events.

Most of the existing research literature on the use of ketamine to treat depressive disorders (unipolar and bipolar) looked at racemic ketamine in the sub-psychedelic dose range given by infusion as a stand-alone treatment. However, ketamine is a versatile tool that can be used in a variety of ways. Here we articulate three paradigms for ketamine treatment: biochemical, psychotherapeutic, and psychedelic. These approaches differ from each other in the ketamine dose, frequency, and intention for the ketamine session. We used three composite clinical vignettes to illustrate how these paradigms could be utilized in treating bipolar patients with refractory depression, noting that different individuals may be well suited to different treatment strategies. Although our discussion focused on ketamine for patients with bipolar disorder (type 1), these paradigms could be applied more broadly to the field. It is also worth noting that the paradigms are discussed separately for the sake of clarity, but in reality, there is some overlap between the paradigms, e.g., a patient who receives ketamine in the psychedelic dose range also benefits from the biochemical effects of the medicine and also receives substantial psychotherapeutic support before, during, and after the medicine session.

One of the challenges in ketamine treatment is that beneficial effect appears to be temporary, especially if one focuses on the “biochemical” effect alone (70, 71). However, in our years of clinical practice, we have come to believe that ketamine and psychotherapy are synergistic and can potentiate each other (as described in our vignettes). It appears that the beneficial of ketamine treatment can be extended in some patients by combining conventional pharmacotherapy, ketamine, and psychotherapy. The addition of psychotherapy can add a space for psychological exploration, relational healing, and learning new skills. We hope to reduce the chance of ketamine dependence by offering ketamine in combination with other interventions.

Finally, we acknowledge that this article contains observations from our clinical practices combined with conceptual ideas. It is important to remember that there is a circular and symbiotic relationship between observations from the field and experimental studies, which continuously inform the other. We hope that this article will inspire other clinicians and researchers to broaden their understanding of ketamine treatment and undertake new research studies.

## Data Availability Statement

The datasets presented in this article are not readily available because we used composite vignettes to protect patient privacy.

## Author Contributions

RB conceived of and wrote the bulk of this article, with input from CY and GB. CY and RB created the list of references. All authors edited the report and approved the report before submission.

## Conflict of Interest

CY was employed by the company Valis Bioscience. CY declares they hold no commercial stake in any product related to racemic ketamine. The remaining authors declare that the research was conducted in the absence of any commercial or financial relationships that could be construed as a potential conflict of interest.

## Publisher's Note

All claims expressed in this article are solely those of the authors and do not necessarily represent those of their affiliated organizations, or those of the publisher, the editors and the reviewers. Any product that may be evaluated in this article, or claim that may be made by its manufacturer, is not guaranteed or endorsed by the publisher.
